# Enabling Modular Robotics with Secure Transducer Identification Based on Extended IEEE 21450 Transducer Electronic Datasheets

**DOI:** 10.3390/s23052873

**Published:** 2023-03-06

**Authors:** Tobias Mitterer, Christian Lederer, Hubert Zangl

**Affiliations:** 1 Sensors and Actuators Department, Institute of Smart System Technologies (IST), University of Klagenfurt (AAU), 9020 Klagenfurt am Wörthersee, Austria; 2 CISC Semiconductor GmbH, 9020 Klagenfurt am Wörthersee, Austria; 3 Silicon Austria Labs (AAU SAL USE Lab), 8010 Graz, Austria

**Keywords:** transducer identification, logical sensor integration, security, wireless transducer

## Abstract

In robotics, there are many different sensors and actuators mounted onto a robot which may also, in the case of modular robotics, be interchanged during operation. During development of new sensors or actuators, prototypes may also be mounted onto a robot to test functionality, where the new prototypes often have to be integrated manually into the robot environment. Proper, fast and secure identification of new sensor or actuator modules for the robot thus becomes important. In this work, a workflow to add new sensors or actuators to an existing robot environment while establishing trust in an automated manner using electronic datasheets has been developed. The new sensors or actuators are identified via near field communication (NFC) to the system and exchange security information via the same channel. By using electronic datasheets stored on the sensor or actuator, the device can be easily identified and trust can be established by using additional security information contained in the datasheet. In addition, the NFC hardware can simultaneously be used for wireless charging (WLC), thus allowing for wireless sensor and actuator modules. The developed workflow has been tested with prototype tactile sensors mounted onto a robotic gripper.

## 1. Introduction

**Figure 1:**
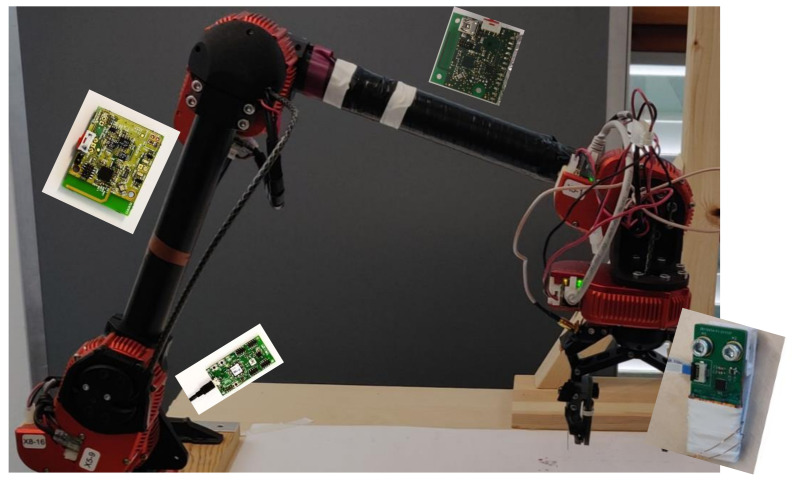
Illustration of a modular robot system with multiple different sensors and actuators which need to be connected. They should be easily interchangeable in the concept of modular robotics.

Current trends in robotics predict an increase in the number of sensors and actuators mounted onto a robot system to be used in different tasks. These transducers could be interchanged to switch the robot system’s current task. Integration of new transducers can be an arduous and time-intensive task, as each could have its own method of connecting and transmitting commands and measurements. In order to reduce the load when integrating such transducers and ensure that the transducer seen in the system corresponds to the currently used transducer when using wireless connections, a secure and fast method for identification and integration is needed. To guarantee a correct identification of the sensor to the system, near field communication (NFC) offers the best functionality, as the wireless transducer can simply be placed onto an NFC gateway. In this manner, a visual verification can be performed, and a key exchange for common secret computation can also be performed without the possibility of interference by third parties. Using NFC additionally enables the system to charge the wireless transducer for the upcoming measurement tasks via wireless charging (WLC). An example of various sensors and actuators which need to be connected to a robot system is illustrated in Figure 1.

Security for transducers connected to wireless sensor networks (WSNs) has been investigated in-depth in the literature. Reviews on the security of WSNs can be found in [1,2,3], whereas an overview on security issues is given in [4,5]. Another survey on security in WSNs is given in [6], where static and dynamic WSNs are compared. Regarding the use of NFC or radio frequency identification (RFID) for transducers [7], a sensor using NFC to detect changes to ethanol mixtures has been developed. In [8], location information of moving sensors is identified using RFID. Regarding sensor identification, in [9], fully passive sensors are identified and powered using RFID. Low power constraints in WSNs are addressed in [10], which compares existing energy harvesting technologies for WSNs. Ref. [11] gives an example of a WSN which is able to communicate with a large range of different transducers. In [12], a robot system where multiple sensors are used in conjunction is investigated. An inclusion of security aspects in electronic datasheet standards such as Institute of Electrical and Electronics Engineers (IEEE) 1451.0 transducer electronic datasheet (TEDS) has been investigated in [13], where a management information base (MiB) is used in conjunction with TEDS. Additionally, in previous work [14], the security in industrial wireless sensor networks (IWSNs) in conjunction with IEEE 1451.0 TEDS has been investigated. An example of using IEEE 1451.0 in conjunction with the International Electrotechnical Commission (IEC) 61499 standard to handle the logical integration of transducers can be found in [15,16,17]. A general investigation and survey into the security of wireless networks has been undertaken in [18], and an in-depth survey of eavesdropping in wireless networks from a security-reliability trade-off perspective is detailed in [19]. A more specific investigation into potential security risks in WSNs is detailed in [20], where the important differences in security for WSNs have been summarized. A review on current cyber attacks related to WSNs is presented in [21], which also surveys attack design and attack detection from the domain of system control with so-called networked control systems. A detailed analysis of security in conjunction with networked control systems, including an implementation for such a system, is presented in [22].

In this work, a concept is proposed to include security aspects in the IEEE 1451.0 TEDS standard with a focus on secure and fast identification of new transducers in a system. The concept results in a workflow starting from the initial configuration and identification to being used in measurement scenarios. To validate the proposed concept, a robotic use-case is demonstrated, where multiple iterations of prototypes of tactile capacitive sensors are mounted on a robot gripper and used in pick-and-place tasks. In the validation scenario, the tactile sensors are nodes that are autarkic low-power wireless nodes which also support NFC for data transmission. The main contribution of this work is the key exchange via NFC, allowing for a secure way to physically identify the wireless sensor node and match it to its digital representation in the system, while also allowing for the exchange of keys in a secure manner, reducing the risk of outside interference in the transmission. Therefore, the security concept developed in a previous paper has been adapted and improved upon in the aspect of identification of sensor nodes.

## 2. Materials and Methods

### 2.1. System Description

**Figure 2:**
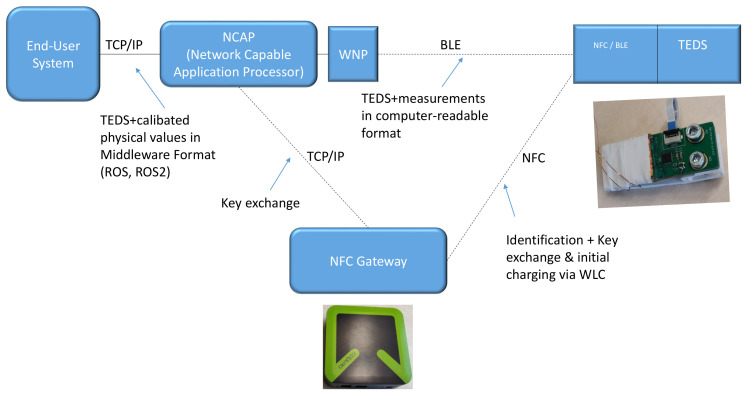
Overview of the system used to connect the sensor to the robot.

In the proposed workflow, an extension to the IEEE 1451.0 TEDS is used to handle the identification of new transducers and exchange of security information. The security extension to the TEDS standard is an adaptation of the one proposed in [14]. For the identification, an electronic datasheet in the TEDS format is prepared on the transducer during development, containing pertinent information such as a universal unique identifier (UUID), calibration coefficients, how many and which sensors and actuators are on the transducer and a signature enabling the base station to verify the trustworthiness of the received information. The network capable application processor (NCAP), as the base station, handles the configuration of new transducers to automate logical integration and establish trust with the new transducer. Transducers connect via the wireless network processor (WNP) using wireless protocols and NFC for data exchange and identification, respectively. The end user interacts with the NCAP, requesting either measurements from sensors or sending commands to actuators on the connected transducers. An overview of what such a system looks like is given in Figure 2.

### 2.2. Electronic Datasheet Security Extension

The TEDS security extension is based on the one proposed in [14], where fields for an industrial use-case, such as a required verification for an external calibration lab, have been included. In the proposed security extension, all fields from 1 to 100 are mandatory fields, with fields from 100 to 255 reserved for manufacturer usage. The implementation is visualized in Table 1. Based on this security TEDS, the new field “UsedSecScheme” has been added to determine if verification of both the transducer and base station has been completed and if either the overall communication or only the transmitted data are encrypted. The preliminary defined options are shown in Table 2. Another adaptation is the change in the previous field “UsedEncAlg” to “UsedVerAlg”, as the algorithms listed herein are to compute and use public and private key pairs mostly for verification via signatures, with its fields detailed in Table 3. To determine which encryption scheme is used to encrypt either communication or only the transmitted data, the field “UsedEncAlg” has been added, with its fields detailed in Table 4. The changes enable a greater adaptability to different scenarios and also to define verification and encryption schemes used independently from one another. The manufacturer-specific fields have been adapted to the robotic scenario, which is shown in this work, in which a public key and a signature of both the transducer and the base station is needed to both verify each other and to compute a common secret.

In Table 3, options for algorithms used to compute private–public key pairs, which can be used to compute and verify signatures, are given and the fields are reserved for future use. The computed key pairs can further be used to either directly encrypt communication or to safely compute symmetric keys for the encryption of communication. In Table 5, options for the used hashing algorithm in combination with the used encryption scheme are given, with a few often used algorithms already defined. The rest can be added as needed into the manufacturer-reserved fields.

### 2.3. Initial Configuration

**Figure 3:**
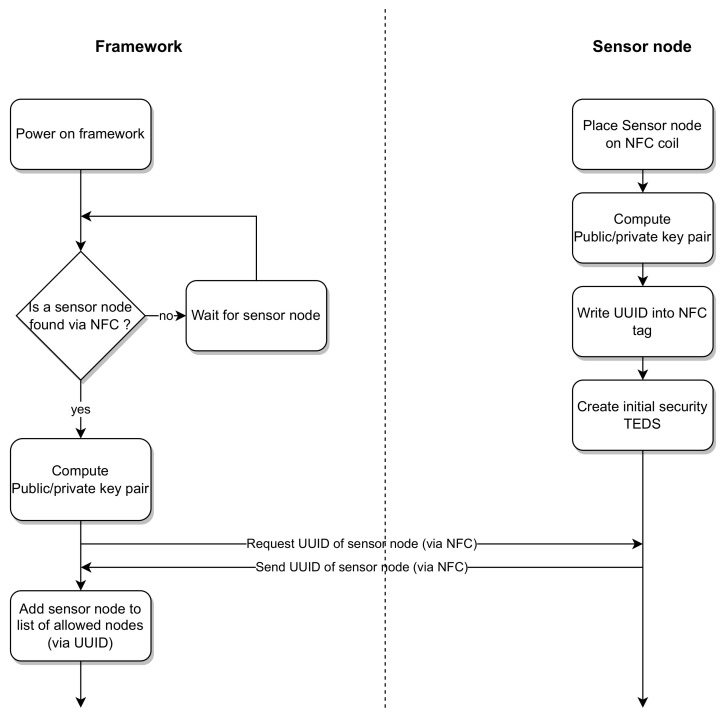
Initial identification workflow for the new transducer.

To prepare a new transducer, it must undergo an initial configuration after development. In this configuration, calibration, security and identification are handled. The NCAP is in a restricted whitelist mode and only allows trusted transducers to be added to the WSN. When a new transducer is to be added to the WSN, it is placed on the NFC Gateway, where it is initially charged and exchanges its UUID with the NCAP. After receiving the UUID, the NCAP adds it to its own whitelist, allowing for wireless communication with this transducer. Furthermore, both the NFC Gateway and the transducer possess a hardware secure element, in which a private–public key pair is generated and stored. On the transducer side, an initial electronic datasheet is created, which includes information such as the UUID of the transducer, its public key and calibration information pertinent to the sensors on the transducer. Figure 3 gives an overview on how the identification of a new transducer is performed.

**Figure 4:**
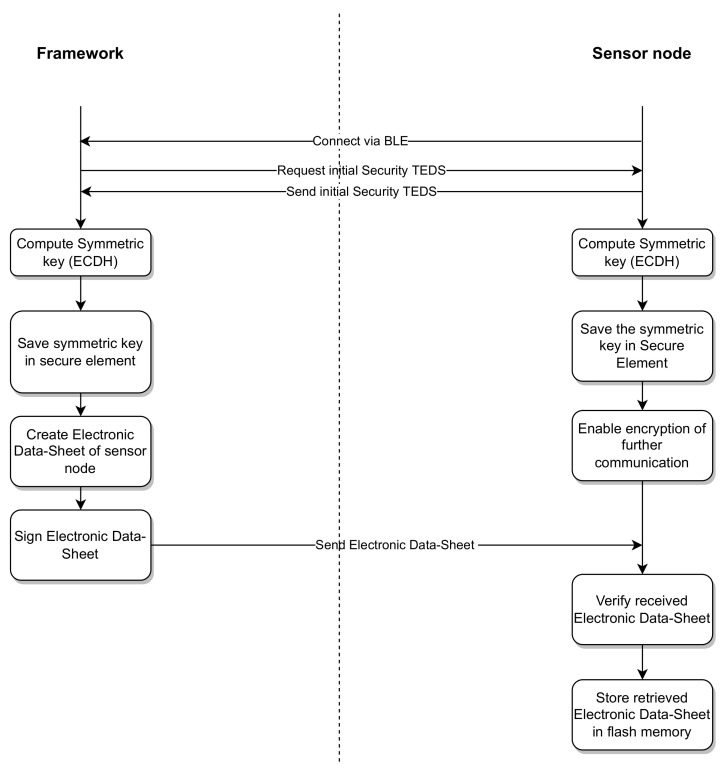
Security workflow for the new transducer.

After the new transducer has been identified to the system, a connection via Bluetooth low energy (BLE) can be established, as illustrated in Figure 4. To complete the initial configuration, the NCAP requests the initial security TEDS from the transducer and transmits its own public key to the transducer in the request. Utilizing the exchanged public keys, both sides compute a symmetric advanced encryption standard (AES) key using the elliptic curve Diffie–Hellman (ECDH) algorithm. This enables them to communicate via the wireless connection in a secure manner. The symmetric key can then be exchanged between different NCAPs. The NCAP retrieves an electronic datasheet created for this transducer type during development and adds calibration and security information. The created electronic datasheet is then signed by the NCAP and transmitted to the new transducer via the wireless connection. On the transducer side, the electronic datasheet is verified and, after successful verification, stored in the flash memory of the transducer.

### 2.4. Measurement Operation

**Figure 5:**
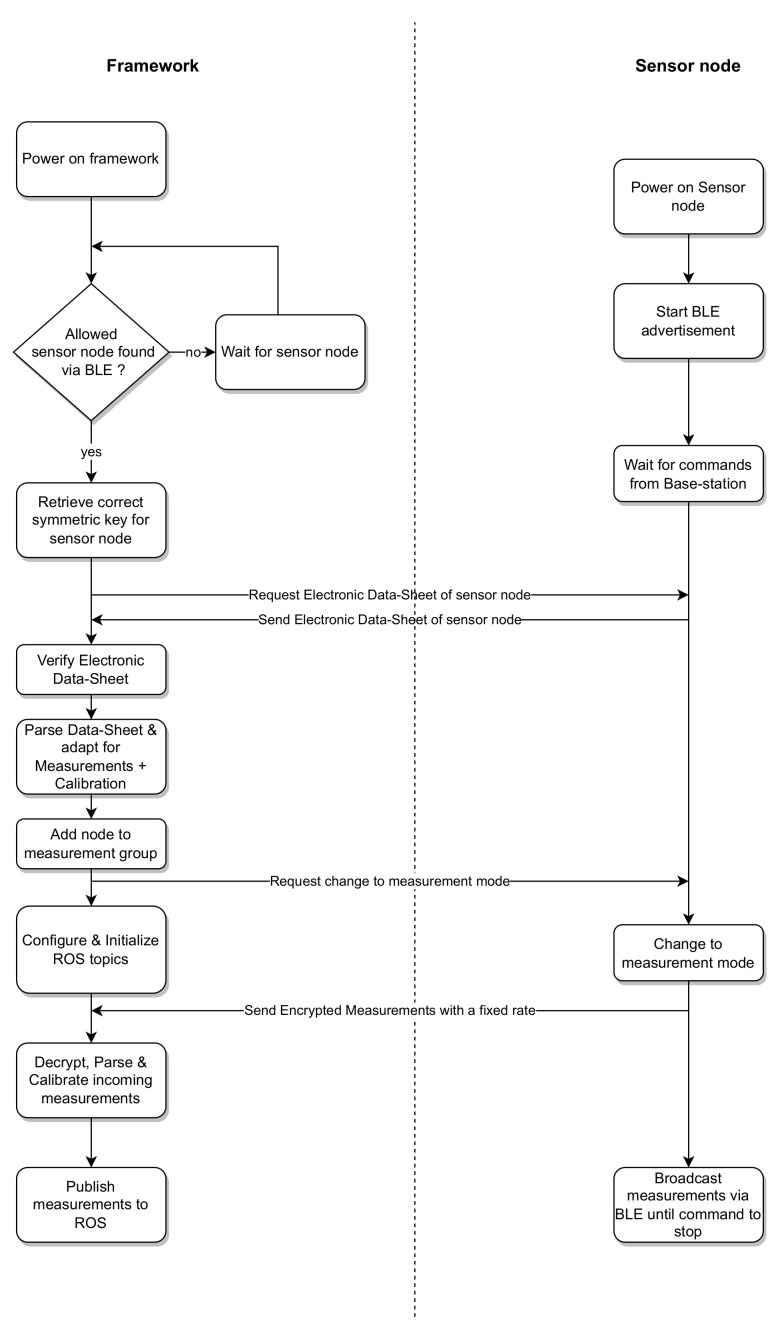
Measurement workflow for the new transducer.

For the operation phase, when a new transducer has not been identified to the system yet, an identification, as illustrated in the beginning of Figure 3, has to be performed. After identification, it can connect to the system via wireless protocols as shown in Figure 5. On the base station side, if a symmetric key for the transducer is available, it is used to encrypt further communication with the transducer. As a next step, the electronic datasheet of the transducer is requested and if no symmetric key is available, the base station’s public key is included in the request. This allows for the computation of a new symmetric key on both sides using the ECDH algorithm. The base station verifies the authenticity of the electronic datasheet and the transducer, respectively, using the signatures inside the electronic datasheet. After trust has been established between both parties, the NCAP uses the information regarding sensor channels and calibration information to configure its interface to be able to correctly interpret incoming measurements from this transducer. When all transducers needed for the task have been added to the system, they are grouped in a measurement group, allowing the NCAP to initialize a measurement. Additionally, the NCAP creates respective robot operating system (ROS) publishers and subscribers, forwarding measurements into the ROS and allowing for actuators to be controlled via the ROS. After and between measurements, the WLC gateway allows for the wireless transducers to charge again to be used for further tasks.

## 3. Results

**Figure 6:**
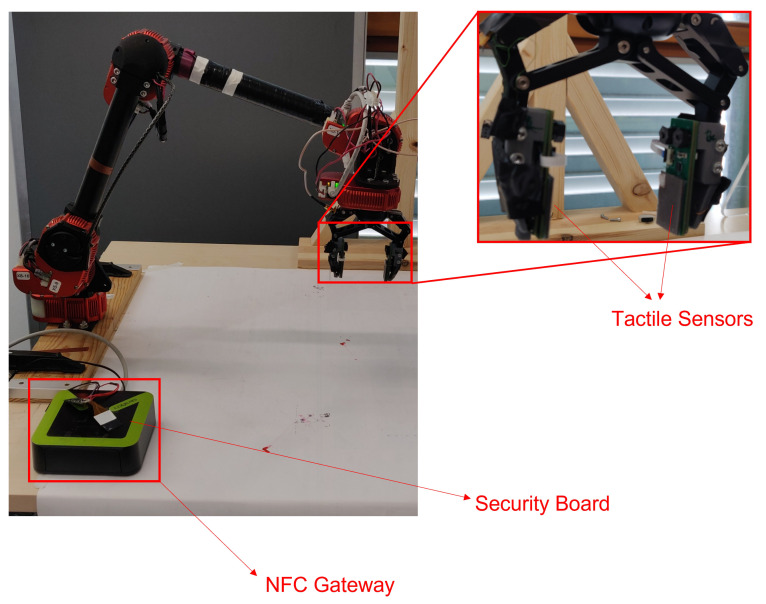
Used robot system and base station, with the sensors mounted in the robot gripper.

**Figure 7:**
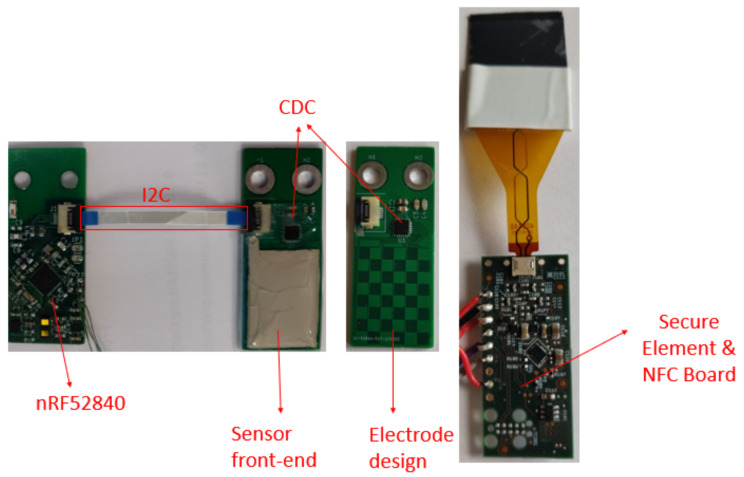
Used modular capacitive sensor board consisting of a microcontroller, a sensor and a security part.

The developed workflow was demonstrated with a modular series elastic 5-DoF arm with a two-fingered cable gripper by HEBI Robotics [24], with tactile sensors used for the gripper, while the framework running the base station for the sensors was an NFC Gateway, as illustrated in Figure 6. The used finger sensor consisted of three modular boards connected via an inter-integrated circuit (I2C), as illustrated in Figure 7. The boards were a microcontroller board with a Bluetooth antenna and an nRF52840 microcontroller, the sensor board consisted of a tactile sensor pad, an electrode design and a AD7147 capacitance to digital converter (CDC) chip. The third board was the security board, consisting of an SE050 secure element, the NFC tag NHS3152 and the wireless charging power receiver PC9431. The NFC gateway on the other side contains an NFC reader PN7362 and an i.MX 6 UltraLite Applications Processor. This setup allows the establishment of a communication link via NFC and furthermore a power transfer from the gateway to the sensor according to the technical specification [25].

In the initial configuration step, both tactile finger sensors were identified to the NCAP using the NFC connection, where their 10-Byte-long UUID was sent to the NCAP. Then, the NCAP requested the initial security TEDS via NFC and added its own public key to the request. On the tactile sensor side, the initial security TEDS, containing its own public key, was sent to the NCAP and a symmetric 128-Bit-long AES key was computed using the ECDH algorithm and Public Key Cryptography Standard #7 (PKCS7) padding. The symmetric key was then stored inside the secure element and used to further encrypt wireless communication. The curve used as a base for the ECDH algorithm was the elliptic curve P-256 with a key length of 256 Bit. On the NCAP side, after the initial security TEDS was retrieved, it was added to a prepared IEEE 1451.0 TEDS, which in turn was signed by the NCAP for each tactile finger sensor. The signed electronic datasheets were then sent back to each tactile finger sensor, where they were stored in flash memory. On the NCAP, the symmetric AES key was also computed using the ECDH algorithm and stored in a secure key storage for each tactile finger sensor. After configuration and identification, the tactile finger sensors were allowed to communicate with the base station using the BLE wireless protocol, where the communication was encrypted with the previously computed symmetric AES key. Additionally, to test the workflow, if the NCAP on which the initial configuration was performed was not the same NCAP that was used for the measurement task, the NCAP requested the TEDS from each tactile finger sensor after connecting via BLE. Using the security information stored in the electronic datasheets, the NCAP verified the authenticity of the TEDS and the trustworthiness of the tactile finger sensor. After both tactile finger sensors were connected to the system and trust was established, a measurement group containing the UUIDs of both transducers was created and the system started a measurement using this measurement group. In the system, ROS topics for each sensor and actuator on both tactile finger sensors were created and supplied with measurements from the transducers. The ROS topics were then used to supply the robot system with up-to-date information on whether an object had been grasped and how good the grasp quality was. This information was then further used in a pick-and-place scenario to help the robot to improve the grasp of the used objects in the scenario. To further test the applicability of the proposed workflow, multiple iterations of these tactile finger sensors with updates to the sensing front-end and also the computation firmware were performed and each iteration was tested in the scenario. An analysis of the tests showed that the proposed workflow works as intended, with average integration and identification times of 10 s and only minor changes to the electronic datasheets needed to account for the differences between prototype iterations.

## 4. Discussion

In this work, a workflow has been discussed on how new wireless transducers can be securely added to a system using electronic datasheets. In this workflow, a transducer is identified by a system using an NFC connection, while it is initially charged using WLC. This establishes trust between the transducer and the system and allows for a visual verification of the physical transducer with its logical representation in the system. To secure the communication between the transducer and the base station, a key exchange, with public keys embedded in an electronic datasheet, is executed either directly through NFC or later through the established wireless connection. The exchanged keys allow for the computation of a shared secret, e.g., via the ECDH algorithm. The workflow has been verified on a use-case of new prototype tactile transducers mounted on a robot gripper platform. In the use-case, it could be shown that the proposed workflow is applicable to robotic applications and that a fast and secure identification and logical integration of new transducers can be ensured. Additionally, it could be shown that due to the NFC identification process in concord with the electronic datasheets, no errors during the logical integration of a new iteration of the used prototype sensors occurred. During the tests using the prototypes of the capacitive wireless sensors, some limitations of the used communication and middleware protocols became apparent. As conventional BLE only supports 20 simultaneous connections, the number of devices in the WSN would be limited. This limitation could be circumvented by using custom or proprietary wireless protocols based on the BLE physical stack, e.g., [26], where hundreds of wireless sensor nodes could connect to a WSN based on BLE. With ROS as the used middleware, a problem can occur when there are too many active ROS topics and insufficient hardware to support it. This can result in increasing lag in the forwarded measurements and inconsistencies in the measurements. A further point which could be improved upon is the central storage of the AES keys in the hardware secure element on the NCAP base station, as these would need to be transmitted to a new base station in case of multiple base stations being needed. Another point to be improved upon is that for new sensor nodes, the identification currently needs to be triggered via a web interface, which could be changed to be interrupt based to further reduce possible user errors and interactions. Apart from the above-mentioned issues, the workflow worked as expected with an average identification and key exchange time of 10 s per sensor node, which is faster than other approaches such as IEEE 802.11 with a pre-shared key, where the passphrase must be prepared and inserted manually by the user.

The security scheme in the proposed workflow consists of first identifying new sensor nodes via NFC and adding a visual confirmation that the wireless sensor node you see in the system is the one before you. Then, the public–private key pairs are computed via an elliptic curve and stored in hardware secure elements both in the sensor node and the base station. The public keys are exchanged via the NFC link and a symmetric AES key is computed using the ECDH. The computed AES keys are stored inside the hardware secure elements. Then, an electronic datasheet is created, supplied with the public keys of both the sensor node and the corresponding base station, and signed by the base station to circumvent malicious alterations to the information inside. The electronic datasheet is then either directly transmitted via the NFC link or via the BLE connection which was established after identification according to user choice. After this first initialization step, each time the wireless sensor node connects to the base station, the communication is encrypted with the respective AES key. As each wireless sensor node is initialized separately, each sensor node has its own AES key when communicating with the base station. This security scheme has been analyzed using the identified potential security issues and requirements regarding security in WSN protocols in [20]. The security requirements of confidentiality, integrity and availability can be seen as fulfilled, as the data are encrypted for each node separately with end-to-end encryption, where the node only reacts to requests from the verified base station. With regards to the identified potential security risks in WSNs, the architecture with one central base station, no communication between sensor nodes themselves and communication always being triggered by the base station circumvents many avenues of attack for external attacks. The secure key exchange, key storage in the hardware secure elements and different encryption keys for each sensor node further reduce the possible options for internal attacks. The sensor nodes themselves may still be susceptible to distributed denial of service (DDoS) or similar attacks as long as they are not actively connected to the base station, owing to using the BLE wireless protocol for data transmission.

Current approaches to add new wireless sensor nodes to a measurement system in a secure manner are, e.g., using and updating a whitelist, where for each new node a UUID has to be provided to the system for initial identification and the node has to be physically marked in a way that it can be clearly identified and matched to its digital representation for each measurement setup. This is to allow for correct placement of the sensor node. The security protocols for encryption and authentication need to be configured on both the base station and the sensor node and are typically the same for all sensor nodes in a measurement setup. When comparing, e.g., the pure whitelist approach, to the NFC and electronic datasheet-based approach proposed in this work, a distinct reduction in the effort and time required for sensor identification and integration could be observed, as the UUID of a new sensor node is transmitted via NFC. Preliminary tests when using a whitelist in comparison to using the proposed NFC-based approach determined that whitelisting takes on average about 5 s longer than the NFC-based approach. Additionally, as the electronic datasheet is tightly bound to the used security scheme and the sensor node itself, a fast authentication of the sensor node can be executed. As the used security scheme for each sensor node is defined in the electronic datasheet and encryption is performed individually for each node, different security protocols could also be used in the same system to allow for an optimization of the WSN security on an individual basis. In the test implementation of the workflow, elliptic curve cryptography (ECC) was used for authentication and key computation in the ECDH algorithm for encryption, and AES was used for end-to-end encryption of the communication. These protocols were chosen based on surveys on which protocols are best suited for low power wireless applications, where one such survey can be found in [27].

## 5. Conclusions

This paper aims to develop a workflow on how secure identification can be established in modular robotics. The work discusses how NFC and IEEE 1451 electronic datasheets can be used in this context and expands on the existing standard by including security principles. The proposed workflow is verified by applying it on prototypes of wireless tactile sensors which need to be securely connected to a robotic gripper for pick and place tasks in a research project. In conclusion, the goals set in the beginning were reached, as the developed workflow was successfully verified in the robotic use-case scenario.

## Figures and Tables

**Table 1:** Proposed adapted IEEE 21450 Security TEDS Extension from [14]. ©2020 IEEE. Reprinted, with permission, from [14].

Id	Field	Description	Type
1	UsedSecScheme	Type of Security	UInt8
2	UsedVerAlg	Verification Algorithm	UInt8
3	UsedEncAlg	Encryption Algorithm	UInt8
4	UsedHashAlg	Hashing Algorithm	UInt8
5	CA	Certificate Authority	String
6	LastModified	Last Modified	TimeInstance
100	Signature	Signature	String
101	NodePublicKey	Node Pub Key	String
102	SigNodePublicKey	Signature Node	String
103	BaseStationPublicKey	Manuf. Pub Key	String
104	SigBaseStationPublicKey	Signature Manuf.	String
105–255	manufacturer reserved	manufacturer reserved	-

**Table 2:** Options for the field “Type of Security”.

Id	Field	Description
0	NoSec	No security used
1	AsVerification	Verification through Signatures
2	DataEncryption	Only symmetric data encryption
3	CommEncryption	Only symmetric communication encryption
4	AsVerAndData	Signature verification and data encryption
5–128	Reserved	
129–255	Manufacturer reserved	

**Table 3:** Options for the field “used verification algorithm” as proposed in [23]. ©2020 IEEE. Reprinted, with permission, from [14].

Id	Field	Description
0	RSA	Rivest, Shamir and Adleman
1	DSA	Digital Signature Algorithm
2	ECDSA	Elliptic Curve Digital Signature
3	ElGamal	ElGamal Signature Scheme
4	ECDH	Elliptic Curve Diffie Hellman
5–128	Reserved	
129–255	Manufacturer reserved	

**Table 4:** Options for the field “used encryption algorithm” as proposed in [23].

Id	Field	Description
0	AES-128	Advanced Encryption Standard 128 Bit
1	Aes-256	Advanced Encryption Standard 256 Bit
2	RC4	Rivest Cipher 4
3	DES	Data Encryption Standard
4	Blowfish	Blowfish
5–128	Reserved	
129–255	Manufacturer reserved	

**Table 5:** Options for the field “used hashing algorithm” as proposed in [23]. ©2020 IEEE. Reprinted, with permission, from [14].

Id	Field	Description
0	MD5	Message Digest Algorithm 5
1	SHA-256	Secure Hash Algorithm 2-256
2	SHA-512	Secure Hash Algorithm 2-512
3–128	Reserved	
129–255	Manufacturer reserved	

## Data Availability

Not applicable.

## Abbreviations

The following abbreviations are used in this manuscript:

MDPI	Multidisciplinary Digital Publishing Institute
WSN	Wireless Sensor Network
IWSN	Industrial Wireless Sensor Network
WLC	Wireless Charging
NFC	Near Field Communication
RFID	Radio Frequency Identification
I2C	Inter-Integrated Circuit
CDC	Capacitance to Digital Converter
NCAP	Network capable application processor
WNP	Wireless network processor
UUID	Universal unique identifier
TEDS	Transducer electronic datasheet
AES	Advanced encryption standard
ECC	Elliptic curve cryptography
ECDH	Elliptic curve Diffie–Hellman
BLE	Bluetooth low energy
ROS	Robot operating system
PKCS7	Public Key Cryptography Standard #7
IEEE	Institute of Electrical and Electronics Engineers
IEC	International Electrotechnical Commission
MiB	Management information base
DDoS	Distributed denial of service
